# Diverse Strategies to Develop Poly(ethylene glycol)–Polyester Thermogels for Modulating the Release of Antibodies

**DOI:** 10.3390/ma17184472

**Published:** 2024-09-12

**Authors:** Daria Lipowska-Kur, Łukasz Otulakowski, Urszula Szeluga, Katarzyna Jelonek, Alicja Utrata-Wesołek

**Affiliations:** Centre of Polymer and Carbon Materials, Polish Academy of Sciences, M. Curie-Skłodowskiej 34, 41-819 Zabrze, Poland; lotulakowski@cmpw-pan.pl (Ł.O.); uszeluga@cmpw-pan.pl (U.S.); kjelonek@cmpw-pan.pl (K.J.)

**Keywords:** thermogels, sol-gel transition, tandem gelation, polymer degradation, nanoparticles, antibody

## Abstract

In this work, we present basic research on developing thermogel carriers containing high amounts of model antibody immunoglobulin G (IgG) with potential use as injectable molecules. The quantities of IgG loaded into the gel were varied to evaluate the possibility of tuning the dose release. The gel materials were based on blends of thermoresponsive and degradable ABA-type block copolymers composed of poly(lactide-co-glycolide)-b-poly(ethylene glycol)-b-poly(lactide-co-glycolide) (PLGA–PEG–PLGA) or poly(lactide-co-caprolactone)-b-poly(ethylene glycol)-b-(lactide-co-caprolactone) (PLCL–PEG–PLCL). Primarily, the gels with various amounts of IgG were obtained via thermogelation, where the only factor inducing gel formation was the change in temperature. Next, to control the gels’ mechanical properties, degradation rate, and the extent of antibody release, we have tested two approaches. The first one involved the synergistic physical and chemical crosslinking of the copolymers. To achieve this, the hydroxyl groups located at the ends of the PLGA–PEG–PLGA chain were modified into acrylate groups. In this case, the thermogelation was accompanied by chemical crosslinking through the Michael addition reaction. Such an approach increased the dynamic mechanical properties of the gels and simultaneously prolonged their decomposition time. An alternative solution was to suspend crosslinked PEG–polyester nanoparticles loaded with IgG in a PLGA–PEG–PLGA gelling copolymer. We observed that loading IgG into thermogels lowered the gelation temperature (T_GEL_) value and increased the storage modulus of the gels, as compared with gels without IgG. The prepared gel materials were able to release the IgG from 8 up to 80 days, depending on the gel formulation and on the amount of loaded IgG. The results revealed that additional, chemical crosslinking of the thermogels and also suspension of particles in the polymer matrix substantially extended the duration of IgG release. With proper matching of the gel composition, environmental conditions, and the type and amount of active substances, antibody-containing thermogels can serve as effective IgG delivery materials.

## 1. Introduction

In biomedical applications, there is a strong demand for innovative biomaterials with tailored structures and properties. Polymer biomaterials are especially valuable as they can be tailored to meet the unique requirements of individual patients or specific applications. Such customization may involve adjusting the material’s mechanical properties, degradation rate, surface chemistry, and bioactivity to create implants, precisely matching a patient’s anatomy, or drug-delivery systems releasing drugs at controlled rates spanning from days to weeks based on individual requirements.

In tissue engineering and regenerative medicine, injectable in situ formed hydrogels have gained appreciation since they can be used in minimally invasive procedures as systems delivering therapeutic substances (drugs, cells, gene, etc.) in a localized manner [1,2,3,4,5]. The polymer precursors of in situ hydrogels undergo spontaneous gelation through chemical or physical crosslinking, allowing the easy encapsulation of therapeutics during their preparation [6,7,8,9,10]. Among injectable gels, so called thermogels, which undergo gelation solely due to a temperature change, are the most promising ones. These gels, also known as thermoreversible gels or thermogelling materials [11,12], below the critical gelation temperature (T_GEL_), behave as a free-flowing sol. When the temperature exceeds the T_GEL_, provided that a sufficient polymer concentration is maintained, the material undergoes a transformation into a non-liquid gel state. This process is reversible upon temperature lowering.

Block copolymers with a well-established hydrophilic–hydrophobic balance are the most eagerly studied synthetic polymers capable of forming thermogels. In these systems, poly(ethylene oxide) (PEG) has been employed as the hydrophilic component [13], while a hydrophobic block is composed of poly(propylene oxide) (poloxamers) [14] or degradable polyester, namely polylactide (PLA), polyglycolide (PGA), poly(lactide-co-glycolide) (PLGA), or polycaprolactone (PCL) [9,15,16,17,18,19]. The individual polymer chains, when dissolved in water, form micelles of a core-shell or flower-like structure depending on whether the AB, ABA, or BAB copolymer is used. At concentrations well above the CMC, micelles pack into ordered structures or aggregate with an increasing temperature, which leads to gel formation [20]. Poloxamers were thoroughly investigated as thermoreversible gels for drug delivery and tissue engineering. Studies of pure polymers but also with the additions of other polymers, nano-/microparticles, or polymer conjugates show several limitations [4,21,22,23]. Such systems are not sufficiently stable, are characterized by a fast erosion, show low long-term cell viability, and are not hydrolytically degradable [24].

PEG–polyester systems have also been widely used as in situ thermogels due to their FDA-approved biocompatibility, resorbability, and biodegradability into non-toxic products [25]. The gelation of these copolymers, including the time and temperature, degradation rate, and drug-release kinetics, is well documented. These properties are influenced by factors, such as the copolymer molar mass and concentration [13], copolymer composition, ratio of hydrophilic to hydrophobic segments [26], block sequence (ABA or BAB) [27], end group chemistry, and modifications thereof [27,28,29,30]. A proper PEG/polyester ratio is crucial for achieving a thermoresponsive transition from sol to gel [27]. Enhancing the hydrophobicity of the copolymer increases the thermodynamic interaction associated with gelation; therefore, sol–gel transition occurs at a lower temperature and/or concentration. The substitution of PLGA, in PEG–PLGA–PEG copolymers, to more hydrophobic PCL leads to gel formation at lower temperatures [31]. A similar effect can be achieved through modification of the terminal hydroxyl groups in PLGA segments with hydrophobic alkyl chains [30,32]. Additionally, changing the block sequence from ABA to BAB (where A refers to PEG and B to PLGA) lowers the temperature of the sol–gel transition [33]. It can be however noted that an increase in polymer hydrophobicity and its concentration reduce, in turn, the degradation rate [34,35,36,37], which can be related to the drug release [36,38]. For example, the degradation rate decreases as the PGA block is exchanged to more hydrophobic PLA or to PCL.

In the context of medical applications, it is crucial to determine the gelation process in a defined polymer–solvent–biologically active compound system. As an example, the presence of salting-in component, such as NaSCN, in a PEG–PLGA–PEG water solution led to an increase in the T_GEL_ [33], while a salting-out component, like NaCl, resulted in a reduction of the sol–gel transition temperature [39]. The sol–gel–sol transition temperature of PCL–PEG–PCL was also decreased in a saline and glucose solution when compared to an aqueous solution [40]. Additionally, the effect of the drug on gelation and mechanical properties should not be underestimated. It was observed that anti-inflammatory or immunosuppressant drugs of low aqueous solubility led to the syneresis of a poly(ɛ-caprolactone-co-lactide)-b-poly(ethylene glycol)-b-poly(ɛ-caprolactone-co-lactide) (PCLA–PEG–PCLA) hydrogel above the T_GEL_, while the addition of some good aqueous-soluble drugs strengthen network formation [41]. When a therapeutic humanized monoclonal antibody (bevacizumab) was integrated into the PLGA–PEG–PLGA hydrogel, the payload did not alter the gel-formation process but shifted the T_GEL_ to a lower temperature [42].

The above information highlights potential restrictions in the application of PEG–polyesters in certain medical fields. These limitations primarily stem from factors, such as the reversibility of the sol–gel process, inadequate mechanical strength and stability of the gels, and the influence of additives or biologically active payloads on material properties. Moreover, insufficient degradation rates can also impact the release of biologically active compounds. Additionally, personalized biomaterials may require systems, also multi-component ones, for predictable, long-term drug release, which involves loading large amounts of drug.

At present, a prominent research trend focuses on the application of additional chemical crosslinking, alongside physical crosslinking, to create hydrogels with enhanced mechanical properties and slower degradation rates. For example, hydroxyl end groups of PLGA–PEG–PLGA copolymers were modified with itaconic acid, thereby introducing both double bonds and carboxyl groups at the ends of the polymer chain [28,29]. These copolymers formed thermogels that were additionally crosslinked via UV irradiation with the use of camphorquinone [43] or lithium phenyl-2,4,6-trimethylbenzoylphosphinate [44] as photoinitiators. This approach led to gels with enhanced mechanical and hydrolytic stabilities, depending on the quantity of itaconic acid, crosslinking time, photoinitiator used, and polymer concentration. Zhou et al. employed a similar method and prepared a blend of PLGA–PEG–PLGA with its acrylated derivative, which was then used as an ink for 3D bioprinting [45]. Copolymers, with an increase in temperature, formed thermogels with excellent shear thinning properties and rapid elastic recovery and were obtained. After printing, the resulting structure was exposed to photoirradiation, which induced additional, chemical crosslinking due to acrylated derivatives. This process resulted in a stable construct with complex shapes and high shape fidelity.

An alternative approach that may regulate the release of a drug or mixture of drugs involves their encapsulation within nano- or microparticles and subsequently suspending this system within a polymer hydrogel matrix [46,47,48,49,50,51,52]. Encapsulation offers a unique way to protect biologically active substances, especially proteins, from premature degradation. With the presence of the hydrogel matrix, these proteins may achieve an improved therapeutic profile [53]. For example, subunit vaccine components (containing highly purified, immunogenic antigens) were loaded into PLGA nanoparticles and incorporated into a PEG–PCL–PLA–PCL–PEG thermoreversible gel [54]. Such a formulation increased the complex viscosity of the system, lowered the T_GEL_, and minimized the burst release of antigens and adjuvants. Mohammadi et al. [55], in turn, composed a multi-drug delivery platform with the aim of postoperative treatment following ocular surgery. An antibiotic was added directly to the gel, while corticosteroid and hypotensive agents were encapsulated within PLGA microparticles and thereafter loaded into the PEG–polyester gels. By adjusting the hydrophobicity of the gel, it was possible to finely tune the drug release at programmed rates and times.

In this work, our objective was to develop a carrier based on a PEG–polyester thermogelling material, specifically designed to accommodate and release an optimal quantity of model immunoglobulin G (IgG), which may be intended for the treatment of joint and nerve fibrosis. Fibrosis is characterized by the excessive accumulation of collagen-rich fibrotic tissue and can result from inflammation, injury, or diseases, such as diabetes and rheumatoid arthritis. Currently, this field of medicine is dynamically developing to prevent the basic problems related to chronic pain and inflammation, limited mobility, joint degeneration, or nerve damage. Significant progress is evident in the development of treatment targeting fibrotic processes. Recently, it was reported that an engineered humanized monoclonal anti-collagen antibody (ACA) inhibits collagen fibril formation and prevents post-traumatic scarring [56,57]. To ensure the proper delivery of this antibody to the injury site, a delivery platform based on thermoresponsive hydrogels was used (with the antibody loaded into the gel at a concentration of 30 mg/mL) [58]. Based on these studies, our research aimed to develop alternative carriers using degradable copolymers and to conduct preliminary tests of their effectiveness. We optimized various formulations composed of PLGA–PEG–PLGA or PLCL–PEG–PLCL thermogels and tested them for the loading and release of model IgG (with the antibody loaded at concentration of 16.8 mg/mL and 33.6 mg/mL). Simultaneously with the physical crosslinking, used for gel formation, we investigated the synergistic chemical crosslinking of the thermogels to enhance the gel stability and prolong the release of IgG. For this purpose, the hydroxyl end groups of selected PEG–polyesters were modified into acrylates and chemically crosslinked using the Michael addition reaction. We also investigated the release of IgG from thermogels loaded with crosslinked PEG–polyester nanoparticles. The thermogelling behavior, degradation, and release studies were conducted in both water and an NaCl solution to approximate conditions closest to those in the human body. By selecting the appropriate gel system, it was possible to manipulate the degradation time of the copolymers and influence the release time of IgG.

## 2. Materials and Methods

### 2.1. Materials

For the preparation of the thermogel, the following triblock copolymers were purchased from PolySciTech (West Lafayette, IN, USA) (Table 1):

IgG from human serum, lyophilized powder (≥95% (SDS-PAGE)), 2,2′-(ethylenedioxy)diethanethiol (95%), acryloyl chloride (≥97%), and chloroform-d, sodium chloride (BioXtra ≥ 99.5%) were purchased from Sigma Aldrich/Merck (Steinheim, Germany) and used as received. Dichloromethane (DCM) was purchased from Avantor (Gliwice, Poland) and dried over anhydrous calcium chloride, distilled and stored over molecular sieves.

### 2.2. Polymers Modification

The PLGA–PEG–PLGA copolymers (samples **P2** and **P4**) were functionalized with acryloyl chloride. For that purpose, 0.7 g of copolymer **P2** (2.187 × 10^−4^ mol) and **P4** (1.55 × 10^−4^ mol) were dissolved separately in 2 mL of dry DCM. The copolymer solutions were placed in an ice bath and cooled to 0 °C. After that, 50 mg (44.8 µL) and 40 mg (35.8 µL) of acryloyl chloride, corresponding to copolymer **P2** and **P4**, respectively (molar ratio acryl:OH 2:1), were added dropwise to the solutions under vigorous stirring in an argon atmosphere. The vials were then sealed and removed from the ice bath, allowing them to reach room temperature. The reactions were performed for about 20 h. The prepared acryl–PLGA–PEG–PLGA–acryl copolymers were purified via dialysis in DCM (MWCO 1000) for two days, with frequent solvent exchanges, and then dried under a vacuum. The samples were designated as **P2M** and **P4M** and stored in the freezer.

### 2.3. The General Methodology of the Thermogel Preparation

#### 2.3.1. Preparation of Thermogels and IgG-Loaded Thermogels

For the preparation of thermogels for degradation and release studies, the two- and three-component copolymer blends (in a mass ratio of 1:1 or 1:1:1) were dissolved at the desired concentration in a 0.9% NaCl solution. The samples were shaken at a low temperature (2–8 °C) for around 3 days to ensure full dissolution. For thermogels that were additionally chemically stabilized with the Michael addition reaction, a crosslinking agent, 2,2’-(ethylenedioxy)diethanethiol, was introduced into the modified copolymer solutions. Below is an example of the crosslinking of the **2M** and **4M** copolymer blend. To 166.7 mg of the copolymer blend dissolved in an aqueous NaCl solution, 31.6 mg (1.73 × 10^−4^ mol, 28.2 µL) of the crosslinking agent was added. The molar ratio of [SH]:[acryl] in all cases was 1:1. In the case when IgG-loaded gels were prepared, the antibody was dissolved in a copolymer solution with a final IgG concentration of 16.8 mg/mL or 33.6 mg/mL. The glass vials containing the above-mentioned solutions were then placed in an oil bath preheated to 33 °C to trigger the gelation process. At that temperature, the gels formed abruptly and were stabilized for several minutes.

#### 2.3.2. Preparation of Thermogels with Embedded Nanoparticles and IgG

To obtain thermogels with embedded nanoparticles and IgG, two approaches were tested. The first method involved initially creating nanoparticles from **P2M** and **P4M** copolymers, which were then mixed with appropriate amounts of IgG. The obtained system was then suspended in polymer **P3**, used as the thermogel matrix. Specifically, a 0.1% mixture of **P2M** (2.5 mg, 7.81 × 10^−7^ mol) and **P4M** (2.5 mg, 7.81 × 10^−7^ mol) copolymers in 0.5 mL of 0.9% NaCl was prepared and left at 4 °C overnight. Once the nanoparticles were formed, 8.4 mg or 16.8 mg of IgG and 1.12 mg of 2,2′-(ethylenedioxy)diethanethiol (6.14 × 10^−6^ mol, 1 µL) were added to the solution and left for 3 h. Then, 167 mg (3.63 × 10^−5^ mol) of polymer **P3** was dissolved in the solution containing IgG-loaded nanoparticles for 3 days at 2–8 °C. The final polymer **P3** concentration was 25 wt% with a final IgG concentration of 16.8 mg/mL or 33.6 mg/mL. After that time, the obtained solution was preheated to 33 °C, forming a thermogel with suspended nanoparticles.

The second method involved nanoparticle formation in the presence of IgG and then suspending the obtained system in polymer **P3** (used as a thermogel matrix). A solution of IgG in 0.9% NaCl (with a specified amount of IgG) was used as the solvent for the mixture of **P2M** and **P4M** copolymers. As previously, the polymers were dissolved overnight at 4 °C, resulting in a final copolymer concentration of 0.1%. After 12 h of nanoparticle formation, 1.12 mg (6.14 × 10^−6^ mol, 1 µL) of 2,2′-(ethylenedioxy)diethanethiol was added to the solution. Then, 167 mg (3.63 × 10^−5^ mol) of polymer **P3** was dissolved in the solution containing the obtained nanoparticles for 3 days at 2–8 °C. The final polymer **P3** concentration was 25 wt%, with a final IgG concentration of 16.8 mg/mL or 33.6 mg/mL. After that time, the obtained solution was preheated to 33 °C, resulting in the formation of a thermogel with suspended nanoparticles.

### 2.4. Thermogel Degradation

The degradation of thermogels was performed in 0.9% NaCl with samples having a final polymer concentration of 25 wt%. An example of such an experiment is shown for the thermogel formed from a blend of copolymers **P2**/**P4**/**P6** in 0.9% NaCl. A total of 400 mg of copolymers **P2**, **P4**, and **P6** (133 mg each) was dissolved in 1.2 mL of filtered deionized water. The resulting solution was divided into 12 vials, each containing 0.1 mL of the sample. These vials were then placed in an oil bath heated to 33 °C. After the formation of gels, they were allowed to stabilize for a few minutes. Then, each gel was flooded with 2 mL of 0.9% NaCl (pre-warmed to 33 °C) and left in the bath to initiate the degradation process. Each of the vials was sequentially removed from the bath after a specified degradation time, and its contents were analyzed.

### 2.5. The Release of IgG from Thermogels

The in vitro release of IgG was determined for thermogels, chemically crosslinked thermogels, and thermogels with embedded nanoparticles. The IgG-loaded thermogels were prepared according to the procedures described in Section 2.3. After the formation of the gels, they were stabilized at an elevated temperature for several minutes and then flooded with 10 mL of 0.9% saline prewarmed to 33 °C. At designated time intervals, the supernatant was transferred into a separate vial and replenished with an equal volume of fresh 0.9% saline that had been pre-incubated at 33 °C. The amount of IgG released was determined using spectrofluorometric measurements. The in vitro release data were represented as the cumulative drug release percentage over time.

### 2.6. Methods

Proton Nuclear Magnetic Resonance (^1^H NMR). The ^1^H NMR spectra of copolymers in CDCl_3_ were recorded on a Bruker^®^ Ultrashield 600 Plus spectrometer (Billerica, MA, USA) operating at 600 MHz with TMS as the reference.

Gel Permeation Chromatography (GPC-MALLS). Gel permeation chromatography with a Dn-2010 RI differential refractive index detector (WGE Dr. Bures, Berlin, Germany) and a multiangle laser light scattering detector (DAWN EOS from Wyatt Technologies, Santa Barbara, CA, USA) was used to determine the molar masses and molar mass dispersities of the copolymers. The GPC was performed in DMF with LiBr at 45 °C and at a nominal flow rate of 1 mL/min using the following set of columns: Polymer Standard Service (Polymer Standards Service, PSS) GRAM 100 Å, PSS GRAM 1000 Å, and PSS GRAM 3000 Å. The results were evaluated using ASTRA 7 software v.7.3.1.9 (Wyatt Technologies, Santa Barbara, CA, USA). The refractive index increments of copolymers were independently measured as a batch method for **P2** and **P3** copolymer solutions in DMF at concentrations from 0.5 to 8 g/L using a SEC-3010 differential refractive index detector (WGE Dr. Bures, Berlin, Germany) and were used to calculate the average molar masses of the copolymers (dn/dc = 0.032). Copolymer samples for mass measurements after degradation were prepared as follows: gel residues obtained after degradation were immersed in 1 mL of DMF containing LiBr at a concentration of 0.43 g/L. The mixture was then passed through a 0.2 µm PTFE syringe filter.

Viscoelastic measurements. The dynamic mechanical analysis (DMA) was performed using a DMA 2980 TA Instruments analyzer (New Castle, DE, USA). The samples were tested using a compression clamp (plates with a diameter of 15 mm). The thin layer (~micrometers) of copolymer solutions (in H_2_O or 0.9% NaCl, also with the addition of IgG) with concentrations of 10% and 25% was deposited between the parallel plates. The initial static force was applied before the start of the compression mode. The mechanical spectra at 1 Hz (oscillation amplitude of 10 µm) were obtained using a temperature scan rate of 0.5 °C/min. The storage modulus (E′) and loss modulus (E”) were obtained as a function of the temperature. Observations of the gelation process of the samples were carried out on the basis of the temperature parameters of the E′ and E″ peak maxima.

Spectrophotometry measurements. The absorbance of copolymers before and after modifications with acryloyl chloride was measured using the Specord 200 plus ultraviolet-visible spectrophotometer (Analytik Jena, Jena, Germany) in the range of 190–500 nm with a maximum at 265 nm. The copolymers were dissolved in methanol at a concentration of 0.05 wt%.

Spectrofluorimetry measurements. Fluorescence measurements were conducted using the HITACHI F-2500 Fluorescence Spectrophotometer (Tokyo, Japan). The fluorescence spectra were obtained at a temperature of 20 ± 0.1 °C using a cell with a path length of 1 cm. Excitation and emission slits were set at 5 nm and 10 nm, respectively. Samples were excited at 280 nm, which is specific for tryptophan, and emission spectra were recorded within the wavelength range of 300–400 nm. The peak maximum, typically around 332 nm, was utilized for IgG release calculations.

Cryogenic Transmission Electron Microscopy (CryoTEM). Cryo-TEM images were captured using a Tecnai F20 X TWIN microscope from FEI Company (Hillsboro, OR, USA), equipped with a field emission gun operating at an acceleration voltage of 200 kV. The images were recorded with a Gatan Rio 16 CMOS camera from Gatan Inc. (Pleasanton, CA, USA) and processed using Gatan Microscopy Suite (GMS) software v.3.31.2360.0 from the same company. Specimen preparation involved dispensing aqueous copolymer solutions (3 mL) onto grids with holey carbon film (Quantifoil R 2/2; Quantifoil Micro Tools GmbH, Großlöbichau, Germany) and rapidly freezing them in liquid ethane using a fully automated Vitrobot Mark IV blotting device from Thermo Fisher Scientific (Waltham, MA, USA). Following preparation, the vitrified specimens were stored under liquid nitrogen until inserted into a Gatan 626 cryoTEM holder (Gatan Inc., Pleasanton, CA, USA) and examined via TEM at −178 °C. Before use, the grids were activated for 15 s in oxygen plasma using a Femto plasma cleaner from Diener Electronic in Ebhausen, Germany.

Cytotoxicity study. The in vitro cytotoxicity study has been conducted according to the ISO 10993-5 standard [59]. The human fibroblasts WI-38 (CCL-75) were obtained from the ATCC (Manassas, VA, USA). The cells were cultured in Dulbecco’s Modified Eagle’s Medium-high glucose (DMEM) supplemented with 10% fetal bovine serum, 100 U/mL penicillin, and 100 μg/mL streptomycin, and 10 mM HEPES was added to the experimental cultures. The cells were cultivated at 37 °C, in a humidified atmosphere containing 5% CO_2_. To study cytotoxicity, 100 μL of the cell suspension, containing 4 × 10^3^ cells, was transferred to wells of the 96-well plates and cultured in standard medium for 24 h. After 24 h, the medium was exchanged to 200 μL of the medium containing extracts of the polymer or extracts of gel material (at different dilutions). The **P2M**/**P4M** polymer blend was dissolved in DMEM at a concentration of 2 mg/mL, filtered through 0.2 µm syringe filters, and then diluted with DMEM to a final concentration of 2–0.16 mg/mL. The extract of **P2M**/**P4M**/**P6** was prepared as follows: the **P2M**/**P4M**/**P6** gel was prepared as 25 wt% of polymers in 0.9% saline, heated at 37 °C, and then flooded with DMEM under sterile conditions. After 24 h, the extract above the gel was withdrawn and diluted (0×–12,800×) with DMEM. The cells were incubated with the tested solutions for 72 h. Untreated cells were used as negative control and cells treated with 5% DMSO as a positive control. The viability of cells was evaluated with the use of Cell Counting Kit–8 (CCK-8). Absorbance was read at 450 nm (reference: 650 nm) at the Spark 10M (Tecan, Männedorf, Switzerland). The results were analyzed using a one-way ANOVA followed by a Tukey post hoc test. A *p* value of <0.05 was considered statistically significant.

## 3. Results

**Scheme 1:**
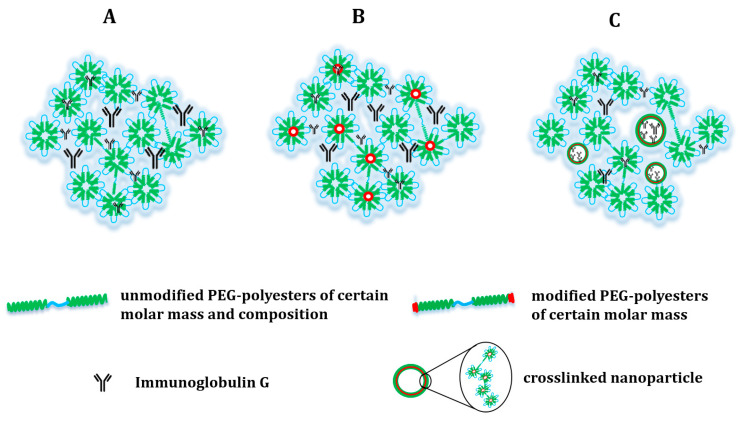
A schematic representation of the thermogel systems used for release of IgG antibody. Thermogels are composed of a two- or three-component blend of unmodified copolymers (**A**), unmodified with modified copolymers (**B**), or of crosslinked nanoparticles embedded in a polymer matrix (**C**).

The objective of this study was to design a thermogel carrier characterized by appropriate stability and degradability, specifically tailored for the controlled release of immunoglobulin G. IgG was loaded into the gels in various quantities, with a preference for higher concentrations, to enable sustained release over time. Such a strategy could find application, for instance, in treating joint or nerve fibrosis with antibodies, thereby reducing the need for frequent injections. PLGA–PEG–PLGA and PLCL–PEG–PLCL were chosen as the building copolymers of thermogels. To meet our requirements for this carrier, we examined a range of thermogels composed of polymeric two- and three-component blends, as well as those that were chemically crosslinked and those containing embedded nanoparticles (Scheme 1).

### 3.1. The Thermogelation of Copolymers

Initially, the primary focus was on assessing the gelation temperature (T_GEL_) of the investigated copolymers and their blends, as well as the mechanical properties of the resulting gels in both water and 0.9% NaCl. Additionally, the impact of IgG addition was examined to determine its influence on the sol–gel transition of copolymers in environments that mimic biological systems, as these conditions are known to affect the T_GEL_ [41].

#### 3.1.1. Thermal and Dynamic Mechanical Properties of One-Component Gels

The T_GEL_ values for one-component gels and a storage modulus (E′–representing the elastic/solid-like property of polymeric materials) are given in Table 2.

**Figure 1:**
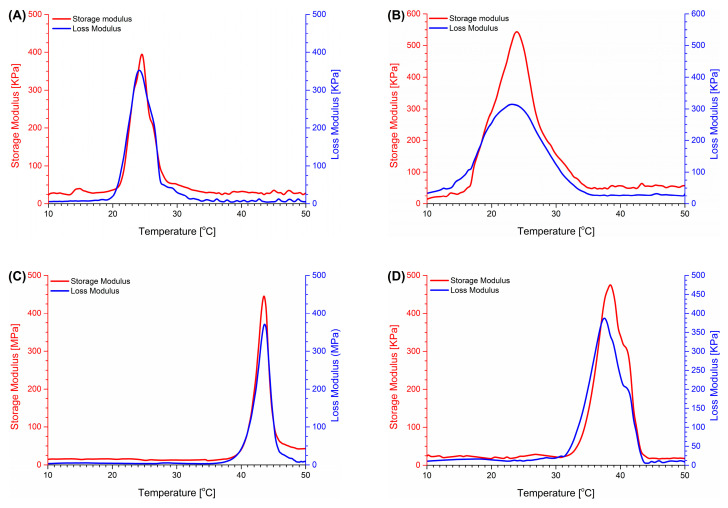
DMA measurement results for exemplifying **P2** and **P4** copolymers at a concentration of 25 wt%: **P2** in (**A**) water and (**B**) 0.9% NaCl; **P4** in (**C**) water and (**D**) 0.9% NaCl (oscillation frequency of 1 Hz).

All PEG–polyester copolymers dissolve well in water and 0.9% NaCl, forming a homogeneous solution at room temperature. As the temperature increases, both the storage modulus and the loss modulus rise rapidly to a maximum value, indicating a sol–gel transition (Figure 1). The gelation temperatures (T_GEL_s) are determined from the maximum values of E′ and E″.

It may be noted that an increase in the polymer concentration in water resulted in a slight increase in the T_GEL_. The gelation temperature of copolymers also depends on copolymer molar mass and the ratio of LA to GA, as similarly observed in the literature [13,26]. An increase in the copolymer molar mass is associated with a higher T_GEL_. For example, for copolymers **P2** and **P3**, despite the same LA:GA ratio (Table 1), an increase in molar mass led to a rise in the T_GEL_ by about 17.5 °C. Conversely, for copolymers **P3** and **P4**, which have the same molar mass and concentration, an increase in the LA:GA ratio from 3:1 to 6:1 also led to a slight increase in the T_GEL_. The storage modulus of gels increased with a higher LA content (e.g., copolymers **P1** and **P2**) or higher molar mass (e.g., copolymers **P2** and **P3**). Furthermore, an increase in the storage modulus can be observed when GA was replaced with CL in the polyester block, as shown for copolymers **P3** and **P6**.

The presence of NaCl in the solution affected the solubility of the copolymers and decreased the gelation temperature compared to copolymers that gelled in water at the same concentration (Table 2). This effect is attributed to the salting-out phenomenon, wherein strongly hydrated ions destabilize the solvated polymer chains, thereby enhancing hydrophobic interactions between them [60]. This behavior also influences the mechanical properties of the gels, resulting in increased stiffness, as indicated by a higher E′ (storage modulus) value.

#### 3.1.2. Thermal and Dynamic Mechanical Properties of Two- and Three-Component Gels

To obtain a thermogel carrier, that forms at a temperature close to physiological conditions (30–37 °C), we used two- and three-component copolymer blends mixed at 1:1 and 1:1:1 ratios, respectively (Table 3). The thermogelation and dynamic mechanical properties of the gels were measured in water and in 0.9% NaCl. It was also crucial to examine how the presence of IgG affects the gelation temperature. Based on the results obtained for one-component gels, the concentration of copolymer blends for gel preparations was chosen to be 25 wt%. This choice was also dictated by the better control of protein release from gels made using higher concentrations, as documented in the literature [61].

**Figure 2:**
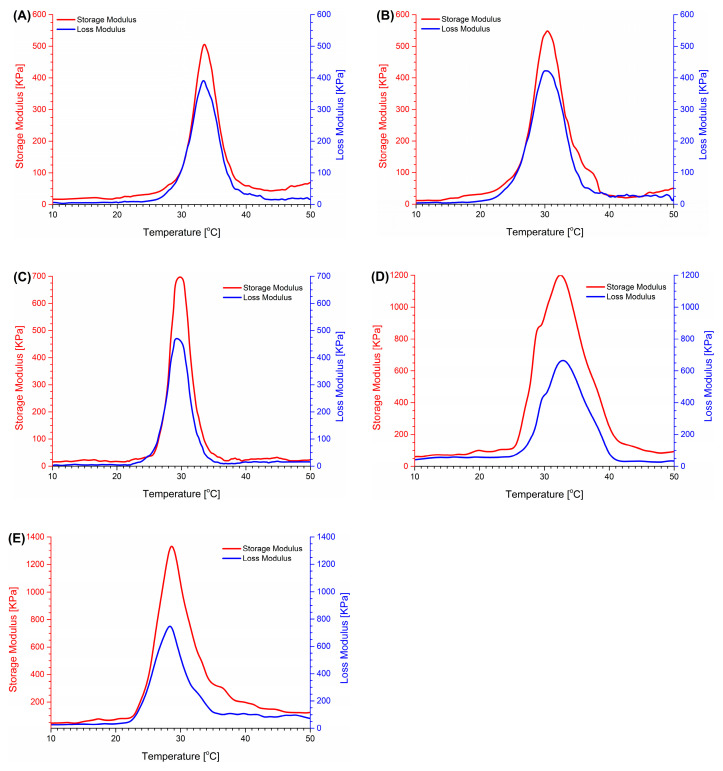
DMA measurement results for exemplifying blends: (**A**) **P2**/**P4** in water, (**B**) **P2**/**P4** in 0.9% NaCl, (**C**) **P2**/**P4** with IgG in 0.9% NaCl, (**D**) **P2**/**P4**/**P6** in 0.9% NaCl, and (**E**) **P2**/**P4**/**P6** blend with IgG in 0.9% NaCl (oscillation frequency of 1 Hz).

As can be noticed in Table 3, the T_GEL_ of the two-component blends in water is averaged over the T_GEL_ of the individual blend components and occurred in the temperature range of 32–35 °C. The T_GEL_ of the copolymer blends in 0.9% NaCl, similarly to that of the one-component system, due to the salting-out effect, is about 3 degrees lower than that measured in water (Table 2, Figure 2A,B). Notably, the E′ values of the gels (from mixtures of copolymers in 0.9% NaCl) significantly exceeded the moduli of the individual components, indicating a more robust structure. Two-component gels formed from blends containing copolymer **P1** are characterized by higher E′ values compared to gels containing copolymer **P2**. The addition of a third component, namely a **P6** copolymer containing CL in its structure (PLCL–PEG–PLCL), also led to a noticeable increase in the E′ value (from 550 kPa for the **P2**/**P4** gel to 1200 kPa for the **P2**/**P4**/**P6** gel), indicating the formation of a more solid gel (Figure 2B,D). The introduction of a CL unit into the system imparts hydrophobicity, which is additionally enhanced by the presence of salt in the solution. Stronger hydrophobic interactions lead, as a consequence, to the formation of a more compact and stiffer gel network.

Based on these data, to prevent the formation of an IgG-loaded gel carrier with an excessively high E′ value, which could affect the medical application and release process of the therapeutic substance, we selected the **P2**/**P3**, **P2**/**P4** and **P2**/**P4**/**P6** systems for additional studies of the effect of IgG on the T_GEL_ and dynamic mechanical properties of the resulting gels. As can be seen, the presence of IgG resulted in the lowering of T_GEL_ values (Table 3, Figure 2C). IgG is a protein with a complex structure, containing both hydrophilic and hydrophobic fragments [62,63,64]. Due to the hydrophilic fragments located on the outer side of the protein [64], the IgG molecule may interact with water molecules. In the presence of IgG, the affinity of copolymers for water molecules diminishes, due to competition between the polymer and IgG for hydration. As a result, the hydrophilic-to-hydrophobic balance in the system is altered. This promotes hydrophobic associations between the PLGA segments of the copolymers (salting-out effect), consequently lowering the T_GEL_. Comparable results were reported by Park et al. [65], who observed that the encapsulation of human growth hormone (hGH) in a biodegradable and thermosensitive poly(organophosphazene) hydrogel lowered the gelation temperature, and Xie et al. [42], who loaded a humanized full-length monoclonal antibody (bevacizumab) onto PLGA–PEG–PLGA hydrogels with similar results.

The storage modulus of the IgG-loaded two- and three component gels was slightly higher than that of the blank material, demonstrating the increased mechanical strength of gels (Table 3, Figure 2C,E). IgG enhances hydrophobic interactions between polyester segments in the copolymers due to the salting-out effect and may also interact with the copolymers’ hydrophobic segments through its own hydrophobic regions [42,62,66].

#### 3.1.3. Thermal and Dynamic Mechanical Properties of Gels from Modified Copolymers

To influence gel stability and mechanical properties and consequently the degradation rate and the extent of antibody release, we tested the synergistic physical and chemical crosslinking of the copolymers. For that purpose, two PLGA–PEG–PLGA copolymers (**P2** and **P4**) were modified with acryloyl chloride (Figure S1). The resulting copolymers were marked as **P2M** and **P4M**, respectively. To verify the success of the modification, ^1^H NMR analysis was performed (Figure S2). After acrylation, three new peaks were observed between 6.0 and 6.44 ppm, corresponding to the protons of the acryl group (peaks at 5.75–5.86 ppm and 6.33–6.44 ppm correspond to protons of the CH_2_= and peaks at 6.00–6.15 ppm to protons of the =CH−). This observation suggests that the hydroxyl end groups were successfully substituted with acryl groups. This conclusion is further supported by the presence of distinctive peaks related to the acryl group in the UV absorption spectra (Figure S3).

Measurements have shown that the hydrophobic modification and chemical crosslinking of the copolymers have an evident effect on the T_GEL_ and mechanical properties of the resulting thermogels (Table 4).

**Figure 3:**
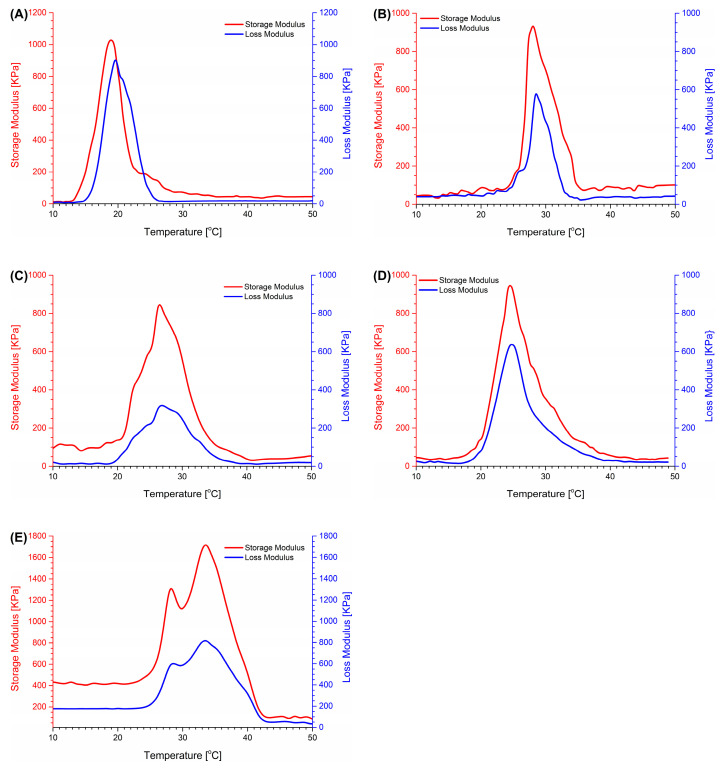
DMA measurement results for (**A**) **P2M** in 0.9% NaCl, (**B**) **P4M** in 0.9% NaCl (**C**) **P2M**/**P4M** blend in 0.9% NaCl, (**D**) **P2M**/**P4M** blend with IgG in 0.9% NaCl, and (**E**) **P2M**/**P4M**/**P6** blend in 0.9% NaCl. Oscillation frequency of 1 Hz.

The study revealed that the incorporation of acryl groups resulted in a lower gelation temperature, reducing it by about 3 °C and 10 °C for the **P2M** and **P4M** copolymers, respectively, compared to the unmodified copolymers (Table 2, Figure 3A,B). This suggest that the affinity for a water solution of NaCl of the block copolymer end groups influences the gelation properties. A similar observation was reported in [45], where the PLGA–PEG–PLGA copolymer (1345:1500:1345; LA:GA 1:1) was modified with acryl groups and used as a bioink. Before modification, this copolymer exhibited high hydrophilicity and solubility, even at elevated temperatures and concentrations. The introduction of the hydrophobic acryl end group led to gelation, although it occurred over a broad temperature range of 37–52 °C.

In the case of the **P2M**/**P4M** blend, its gelation temperature has a value averaged from the values of the individual components, similarly to the results obtained for the gels based on unmodified copolymers (Figure 3C). However, when the third component (copolymer **P6**) was added to the **P2M**/**P4M** blend, two maxima were visible on the temperature sweep plot during the gelation of such a system (Figure 3E). The first maximum may correspond to the gelation of the **P2M**/**P4M** blend, while the second one can be probably related to the presence of copolymer **P6** in the mixture composition. Similarly to unmodified copolymers, the addition of IgG to the blends reduced their T_GEL_ by several degrees, probably due to the competition between IgG and the polymer chain for hydration.

The utilization of dual physical and chemical cross-linking (**P2M**/**P4M** and **P2M**/**P4M/6** gels) enhances the stiffness of the gel, in comparison to their unmodified **P2**/**P4** and **P2**/**P4**/**P6** counterparts, given the resulting increase in the storage modulus (Table 3 and Table 4). This enhanced stiffness arises from the increased network density and stability provided by the combination of permanent chemical bonds and temporary physical interactions.

### 3.2. The Hydrolytic Degradation of Thermogels

**Figure 4:**
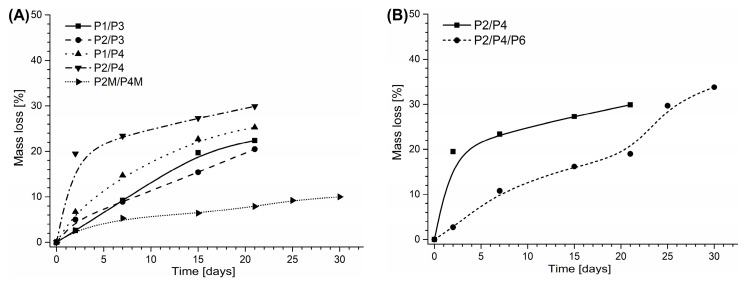
(**A**) Decrease in molar mass during degradation of two-component gels made of non-modified and modified copolymers; (**B**) comparison of molar mass loss during degradation of **P2**/**P4** and **P2**/**P4**/**P6** gels (0.9% NaCl, 33 °C).

Monitoring PEG-polyester degradation is crucial for the controlled release of IgG and its effective use as a delivery system. The degradation process for two- and three-component gels was performed in 0.9% NaCl at 33 °C and proceeded for up to 30 days (Figure 4).

As can be seen in Figure 4A, for all studied two-component gels of non-modified copolymers, the percentage molar mass decrease during 21 days of degradation was between 20% for the **P2**/**P3** blend and 30% for **P2**/**P4**. Over the first 4 days, a leap in the molar mass decrease was evident for the **P2**/**P4** blend, while in later days, the loss was linear. In contrast, for the other two-component gels, degradation was linear over the 3 weeks. The GPC traces of copolymers during 21 days of degradation showed a monomodal distribution for all studied samples (Figure S4). Visual observations confirmed that no changes were noticed in the solutions of all gels during the first few days of degradation. After an average of 5 days, the solutions above the gels became cloudy, while the gels themselves remained clear, indicating that the gel’s degradation process had begun. The gels underwent a slow swelling process, which may have resulted in the formation of new pores in the thermogel and the release of degradation products, causing gradual turbidity of the solutions above the gels. Over the next few days, the gel became looser and began dissolving into the solution, eventually disintegrating into several pieces on the 20th day.

It is known that chemical crosslinking reduces the rate of polymer degradation [29]. As a result, the hydrogels are more stable and degrade more slowly. In our studies, the use of additional chemical crosslinking for the gel formed from modified **P2M**/**P4M** copolymers significantly impacted its degradation (Figure 4A). Comparing the degradation of the **P2**/**P4** and **P2M**/**P4M** blends, a notable slowdown in the process was observed. On approximately the 18th day of degradation, while the **P2**/**P4** gel had already disintegrated, the **P2M**/**P4M** gel remained stable. After 30 days, the decrease in the molar mass of **P2M**/**P4M** was only about 10%. During a visual observation, it was noted that the solution above the gel became turbid, and the gel changed in volume.

It was found that the presence of CL in the gel (**P2**/**P4**/**P6**) resulted in slower decomposition compared to all gels made from the two-component blend. As shown in Figure 4B, the degradation rate of the **P2**/**P4** gel was faster than that of the **P2**/**P4**/**P6** sample. This behavior can be attributed to the hydrophobic nature of the CL block in **P6**, which increases the hydrophobicity of the gel, hindering water penetration into the gel matrix and thus prolonging degradation.

### 3.3. The In Vitro Immunoglobulin G Antibody Release from Thermogels

The developed thermogels were used to investigate the release of a model IgG antibody (Scheme 1). To fine-tune the IgG-release profile, we examined the effects of the hydrogel composition and additional chemical stabilization. We also evaluated the current approach for controlled IgG release, which involved thermogels with embedded nanoparticles and the antibody. Such an approach was described in [46,67], where PLGA nano- or microparticles loaded with IgG were dispersed in hydrogels made of hyaluronan–cellulose or alginate–chitosan. In our studies, the concentration of IgG in all studied thermogel systems was varied (16.8 and 33.6 mg/mL) to adjust the amount of released drug and to assess the feasibility of creating a sustained-release, antibody-based system. To closely mimic physiological conditions, IgG release was conducted in a 0.9% NaCl solution at 33 °C. This temperature is within the range typical for joints [68] and also allows for potential use of the tested system in other areas with similar temperatures where fibrosis may occur.

#### 3.3.1. IgG Release from Two- and Three-Component Gels

**Figure 5:**
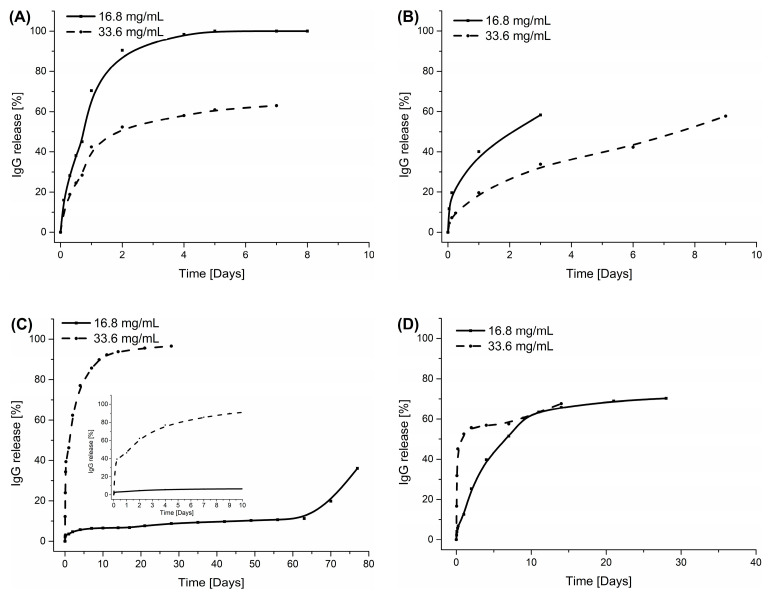
IgG cumulative release from (**A**) **P2**/**P4,** (**B**) **P2**/**P4**/**P6**, (**C**) **P2M**/**P4M**, and (**D**) **P2M**/**P4M**/**P6** blends.

In the literature, the IgG-release profile typically consists of three stages: an initial burst release (phase I), a lag phase (phase II), and a second release phase (phase III) [67]. Both the uncontrolled rapid release associated with peak drug exposure and the lag phase that hinders continuous delivery are undesirable. In light of this, we conducted release studies of the model IgG from gels that were crosslinked both physically (through thermogelation) and chemically (via Michael addition)-specifically, **P2M**/**P4M** and **P2M**/**P4M**/**P6**. For a comparison, similar studies were conducted for counterparts that were crosslinked only physically (**P2**/**P4** and **P2**/**P4**/**P6**). The cumulative release profiles of IgG from the 25 wt% polymeric blends are shown in Figure 5.

Figure 5A shows that approximately 40% of the antibody was released within the first day from the **P2**/**P4** thermogel, regardless of the concentration of encapsulated IgG. As visual observations did not reveal any changes in the solution at that time, the IgG release is likely due to the loosely bound antibody near the interface between the gel and the solution. For a 16.8 mg/mL concentration of encapsulated IgG, the solution above the gel became cloudy after four days, possibly due to the onset of gel degradation. By the fifth day, nearly 100% of the IgG had been released, although the gel had not yet disintegrated. Over the next few days, the gel began to visibly break down and was completely disintegrated by the ninth day, resulting in a highly turbid solution. When a higher amount of IgG (33.6 mg/mL) was encapsulated in the **P2/P4** gel, the release profile showed a slight variation, with an initial burst release on the first day followed by a more gradual release. Additionally, the release process was slowed downed, as until the seventh day, only 60% of the IgG had been released. After that time, the gel disintegrated, which interfered with fluorescence measurements and made further analysis impossible. For both IgG concentrations used, the behavior of the gels aligns with their degradation profile during the initial five days (Figure 4B).

The viscoelastic measurements initially suggested that immunoglobulin enhances the mechanical properties of physically crosslinked gels relative to IgG-free thermogels (Table 3). It should be noted, however, that in further release steps, the presence of IgG accelerates the degradation of the gel, despite identical experimental conditions with regard to the degradation process. This effect is particularly pronounced at higher IgG concentrations compared to gels without IgG.

The addition of a more hydrophobic PLCL–PEG–PLCL copolymer (**P6**) to the **P2**/**P4** gel not only slowed the rate of gel degradation (Figure 4B) but also reduced the rate of IgG release (Figure 5B). At an IgG concentration of 16.8 mg/mL, the thermogel released about 60% of the antibody within four days, after which the gel disintegrated. At an IgG concentration of 33.6 mg/mL, approximately 20% of the IgG was released on the first day, followed by the slow and sustained release of an additional 40% up to the ninth day. In this case, the increased hydrophobicity leads to reduced water uptake and delays the degradation of **P6** gels (Figure 4B). As a result, IgG remains entrapped within the gel matrix for an extended period. Additionally, the gel containing CL units demonstrates greater mechanical stability (Table 3), further contributing to the slower release of IgG. This effect may also be influenced by potential interactions between IgG and the hydrophobic segments of the polymer.

A different IgG-release profile is observed when dual, chemically and physically crosslinked gels are used (Figure 5C,D). Regardless of the IgG concentration loaded, the release from the double-crosslinked gel was extended compared to gels that were only physically crosslinked (**P2M/P4M** vs. **P2/P4** or **P2M**/**P4M**/**P6** vs. **P2**/**P4**/**P6**). Chemical crosslinking enhances the stability of the gels, prolonging both the degradation time and the IgG release, primarily by impeding water penetration into the gel. Additionally, an inverse relationship was observed between the degree of IgG release and its concentration in the gel. For a lower IgG concentration, the release profile from the **P2M/P4M** gel exhibited distinct phases: initially, 5% of IgG was released within one day (Phase I), followed by a lag phase of 8 weeks, maintaining cumulative release at 15% (Phase II). Subsequently, gel degradation led to increased release (Phase III), reaching 40% by the end of the 10th week when the gel completely disintegrated. Conversely, for a higher IgG-loaded concentration, the rapid release of 40% of IgG occurred within one day, followed by gradual release reaching 80% after one week. The gel disintegrated after 27 days with 90% of IgG released (Figure 5C). For the three-component **P2M**/**P4M**/**P6** gel (Figure 5D) a higher concentration of loaded IgG also led to a its faster initial release (about 60% within one day) and gel disintegration as compared to **P2M/P4M**. The burst release of IgG may indicate that some IgG was weakly bound to the gel formed by **P6.** When a lower amount of IgG was entrapped in **P2M**/**P4M**/**P6**, its release was slower, with approximately 65% of the IgG being released gradually over 10 days. The addition of the caprolactone-based copolymer **P6** to the **P2M**/**P4M** gel not only altered the release dynamics but also accelerated the breakdown of the gel (Figure 5C,D, **P2M**/**P4M** vs. **P2M**/**P4M**/**P6**). This may be due to a different gel structure resembling a semi-interpenetrating network, where copolymers **P2M** and **P4M** are crosslinked both physically and chemically, while copolymer **P6** is only physically crosslinked. Additionally, when the weakly bound IgG was released from the **P6** gel, the gel lost further stabilization, creating many pores that allowed water penetration and led to gel disintegration. Finally, chemical crosslinking ensured that the presence of IgG did not accelerate gel disintegration, as it did with physically crosslinked gels. The disintegration time of such gels overlapped with the disintegration time of gels without IgG (Figure 4A). In fact, the presence of a lower amount of IgG in the **P2M**/**P4M** gel even prolonged its breakdown time.

#### 3.3.2. IgG Release from Nanoparticles Embedded in Thermogel

**Figure 6:**
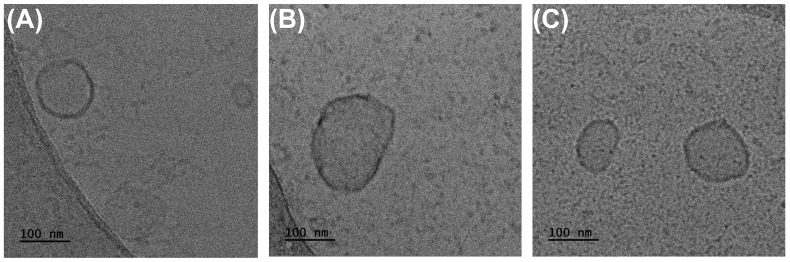
CryoTEM images of (**A**) empty nanoparticles, and representative **P2M**/**P4M** nanoparticles loaded with IgG at a 33.6 mg/mL concentration via (**B**) the diffusion method (IgG_I) and (**C**) entrapment during formation method (IgG_II).

Another approach explored in this work aimed to influence the IgG-release profile by incorporating nanoparticles suspended in the thermogel. This combination of nanoparticles and thermogel could allow for the fine-tuning of release kinetics, as it constitutes a double barrier during IgG delivery. To achieve this, IgG at concentrations of 16.8 and/or 33.6 mg/mL was mixed with polymer micelles prepared from modified **P2M** and **P4M** copolymers. Two methods for incorporating IgG into such systems were tested. The first method involved diffusing IgG into preformed nanoparticles (**P2M**/**P4M**/**P3**/IgG_I). The second method involved entrapping IgG during the formation of the nanoparticles (**P2M**/**P4M**/**P3**/IgG_II). Cryo-TEM images of nanoparticles with IgG and empty nanoparticles are shown in Figure 6.

As depicted in Figure 6, the **P2M**/**P4M** nanoparticles have a relatively round shape and uniform distribution, with sizes of about 100 nm. The formation of nanoparticles from amphiphilic acryl–PLGA–PEG–PLGA–acryl copolymers was based on a self-assembly process driven by hydrophobic interactions of the acryl–PLGA blocks. Based on their sizes, these structures appear to be a form of micellar aggregates. Several aspects might explain this behavior. First, the end-capped hydrophobic acryl groups create a stronger driving force for the assembly of the block copolymers and allow for the formation of crosslinking bridges between micelles. Additionally, the assembly process was performed in water with the addition of NaCl, and as we presented earlier in our studies, the addition of inorganic salts leads to more complex interactions between polymer chains [69]. All this may facilitate the aggregation of micelles into larger assemblies. In contrast to the empty nanoparticles (Figure 6A), a notable concentration of IgG is observed inside the nanoparticles (Figure 6B,C).

The nanoparticles along with the IgG, without separating them from the solution, were then suspended in a **P3** polymer matrix. The residual IgG, which may not have penetrated the interior of the nanoparticles, was subsequently encapsulated within the **P3** thermogel.

**Figure 7:**
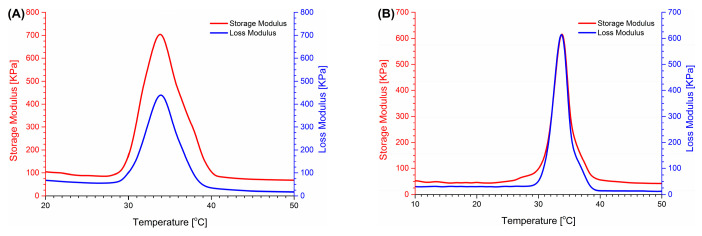
DMA measurement results for (**A**) **P2M**/**P4M** nanoparticles suspended in **P3** thermogel with IgG (**P2M**/**P4M**/**P3**/IgG_II, 33.6 mg/mL IgG) and (**B**) blank nanoparticles suspended in **P3** thermogel. Storage modulus and loss modulus results were obtained at an oscillation frequency of 1 Hz.

The entrapment of nanoparticles and IgG in the **P3** gel slightly lowered its T_GEL_ compared to the **P3** gel without the suspended particles (Table 2, Figure 7A). In contrast, when comparing the properties of a system with embedded blank nanoparticles to that with IgG, it is evident that the presence of IgG in the nanoparticles slightly enhanced the initial storage modulus of the resultant system (Figure 7A,B).

**Figure 8:**
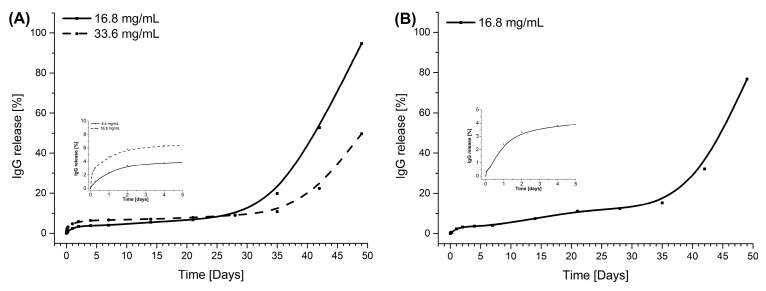
IgG release plots from the (**A**) **P2M**/**P4M**/**P3**/IgG_I (**B**) **P2M**/**P4M**/**P3**/IgG_II system.

The use an approach based on embedding the **P2M**/**P4M** micelles and IgG in the thermogel significantly affected the release model compared to the systems previously presented. Regardless of the technique used to prepare the nanoparticles, the IgG release was prolonged and did not exhibit a phase of abrupt release (Figure 8).

As shown in Figure 8A, a minor concentration dependence can be observed. In both cases, after an initial IgG release of about 5% within the first three days, a gradual slowdown was noted. The duration of this phase was influenced by the initial IgG concentration. For the gel with 16.8 mg/mL of IgG, the final release phase began around the fourth week, with nearly 100% of the IgG gradually released by the 50-day mark. Conversely, for mixtures with a higher IgG concentration of 33.6 mg/mL, the lag phase extended to five weeks. After this period, the release rate increased, but only 50% of the antibody had been released by the 50-day mark.

A more gradual release of IgG was observed for the thermogels containing nanoparticles that were formed in the presence of IgG (Figure 8B). In this case, the IgG release occurred gradually over 35 days, with a cumulative release of 20%. This was followed by a more abrupt release, leading to a cumulative release of up to 80%.

When comparing the two methods used for nanoparticle formation with IgG, it is evident that the second method, which incorporates IgG during particle formation, is more efficient. This is likely due to its higher nanoparticle-loading capacity, in contrast to the method that relies on diffusion-driven IgG loading. As a result, this approach produces a more controlled and gradual IgG-release profile.

Regardless of the method used for entrapping IgG in the nanoparticles, this approach, combined with the stabilization of nanoparticles in the polymer matrix, slowed down the release of IgG. The selection of the **P3** copolymer was highly effective, as it successfully bound the IgG that was not encapsulated within the nanoparticles of the **P2M/P4M** system. Initially, the IgG release occurred from the polymer matrix, followed by gradual release resulting from the disintegration of the **P3** gel, as well as from the release from nanoparticles.

Similar solutions for IgG release have been used where IgG was loaded into PLGA microspheres immersed in an alginate gel [46] or into nanoparticles formed from PCL–b–PLA–b–PEG–b–PLA–b–PCL within a gel matrix of mPEG–b–PCL–b–PLA–b–PCL–b–PEGm [47]. In these cases, as for our system, the use of microspheres or nanoparticles prevented the burst release of IgG. The use of microspheres with a different LA:GA ratio also slowed the release time of IgG by up to seven weeks, as compared to the release of IgG from the polymer matrix alone [46]. Similarly, the release of IgG-Fab from the composites was negligible compared to the rapid release observed from nanoparticles without the polymer matrix [47]. When the polymer concentration in the composite was 15% or 20%, 60% and 40% of IgG-Fab was released within 80 days, respectively. In contrast, more than 90% of Fab-IgG was released from the nanoparticles themselves, not embedded in the polymer matrix, within 35 days.

### 3.4. Cytotoxicity Study

**Figure 9:**
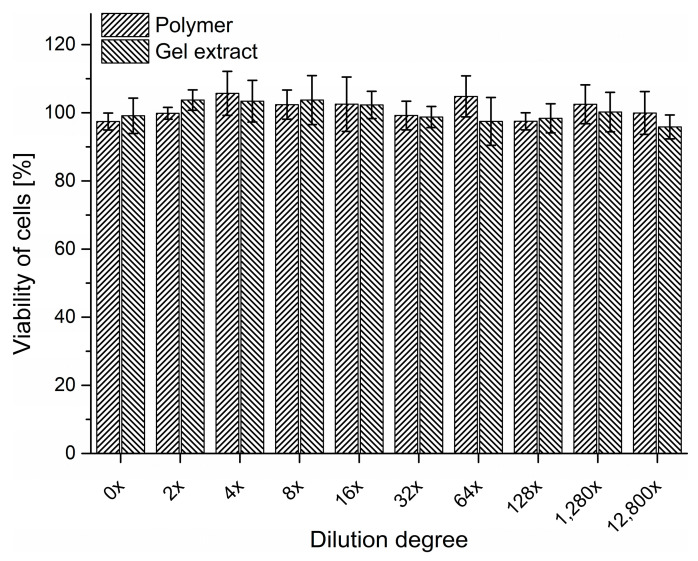
Effect of **P2M**/**P4M**/**P6** polymer and the extract of the **P2M**/**P4M**/**P6** gel on proliferation of normal human cells after 72 h (*p* < 0.05 vs. control group).

To evaluate the in vitro cytotoxicity of the investigated systems, the CCK-8 assay for the representative **P2M**/**P4M**/**P6** polymer and gel made of it was performed. CCK-8 is a sensitive colorimetric technique for the determination of the number of viable cells using WST-8 (2-(2-methoxy-4-nitrophenyl)-3-(4-nitrophenyl)-5-(2,4-disulfophenyl)-2H tetrazolium, monosodium salt) that is reduced by cellular dehydrogenases to an orange formazan product. The amount of the produced formazan is directly proportional to the number of living cells. The results presented in Figure 9 show that the viability of cells was not affected by the presence of both tested materials in the whole studied concentration range, which proves their cytocompatibility.

## 4. Conclusions

In the presented studies, we demonstrate the development of gel carriers designed to deliver an optimal amount of immunoglobulin G (IgG) antibody. The gels were composed of degradable PLGA–PEG–PLGA and PLCL–PEG–PLCL two- or three-component blends, with their formation achieved through physical (thermogelation) or dual physical and chemical gelation. The two-component blends consisted of PLGA–PEG–PLGA copolymers of varying masses and LA:GA ratios, while the three-component blends contained an additional PLCL–PEG–PLCL copolymer. Chemical crosslinking of the copolymers was facilitated by the Michael addition reaction between the acryl-modified PLGA–PEG–PLGA and the crosslinking agent. Additionally, systems of chemically crosslinked nanoparticles from modified PLGA–PEG–PLGA copolymers embedded in a PLGA–PEG–PLGA thermogel matrix were also developed.

The copolymer blends displayed a broad gelation temperature range, from 21 to 38 °C, depending on their composition. The T_GEL_ and dynamic storage modulus of the carriers depended not only on the molar mass or copolymer composition but also on the modification of copolymers with acryl groups, the presence of IgG, and the environment in which gelation occurred. The modification and inclusion of the antibody resulted in a lower T_GEL_, while the storage modulus of the gel increased.

By altering the composition of the polymer carrier, introducing double crosslinking, embedding nanoparticles, or varying the concentration of encapsulated IgG, it was possible to regulate the rate and amount of antibody release.

The modification of the hydroxyl groups in the copolymer allowed for additional chemical stabilization of the gels, thus slowing their degradation and the release of IgG. A similar effect was observed when caprolactone-based copolymers were added to the copolymer blends. The incorporation of IgG nanoparticles into the thermogel matrix resulted in a system that prevented the rapid release of IgG and significantly reduced the release rate.

In therapies requiring longer release times, systems that are dually crosslinked (**P2M**/**P4M** vs. **P2M**/**P4M**/**P6**) or have nanoparticles dispersed in a polymer matrix (**P2M**/**P4M**/**P3**/IgG) appear to be more suitable. The addition of chemical crosslinking (**P2M**/**P4M** blends) prolonged release times up to 27 and 80 days, depending on the concentration of loaded IgG, compared to the solely physically crosslinked systems. The antibody release time from all nanoparticle-based systems was 48 days for both IgG concentrations, while a more gradual profile was obtained for thermogels containing nanoparticles that were formed in the presence of IgG.

To summarize, the results show that antibody-containing thermogels can serve as effective IgG delivery materials, for example, for injection in the treatment of joint fibrosis and nerve tissues, provided that the composition of the gels, environmental conditions, and the active agent and its amount are properly matched. A varied approach to developing various forms of thermogels—blended with encapsulated nanosystems, containing different doses of IgG—may provide a starting point for research on fibrosis, such as arthrofibrosis and nerve scarring. These systems allow for loading of the antibody in high concentrations, potentially avoiding frequent administration of the antifibrotic drug, which typically must be administered regularly [58].

## Figures and Tables

**Table 1:** The characteristics of PEG-polyesters used for thermogel preparation.

Polymer	Manufacturer Number	Sample Number ^a^	Polyester Block Composition	Molar Mass of Blocks [Polyester–PEG–Polyester]
PLGA–PEG–PLGA	AK012	**P1**	LA:GA (1:1)	1000:1000:1000
PLGA–PEG–PLGA	AK024	**P2**	LA:GA (3:1)	1100:1000:1100
PLGA–PEG–PLGA	AK088	**P3**	LA:GA (3:1)	1600:1500:1600
PLGA–PEG–PLGA	AK091	**P4**	LA:GA (6:1)	1500:1500:1500
PLCL–PEG–PLCL	AK108	**P6**	CL:LA (3:1)	1600:1500:1600

^a^ The subsequent numbers in the table indicate the sample symbol appearing in the manuscript.

**Table 2:** Gelation temperatures and storage moduli (values at T_GEL_) of non-modified copolymers in water and 0.9% NaCl.

Polymer	T_GEL_ in H_2_O [°C]		T_GEL_ in NaCl [°C]	Storage Modulus [E′] [kPa]		
	10%	25%	25%	10% H_2_O	25% H_2_O	25% NaCl
**P1**	22.7	24.3	22.0	112.0	247.0	524.0
**P2**	22.3	24.0	23.8	145.0	409.0	588.0
**P3**	40.0	41.5	38.0	140.0	623.0	661.0
**P4**	43.7	43.5	38.7	147.0	434.0	482.0
**P6**	41.5	43.1	21.5	587.0	1162.0	1082.0

**Table 3:** Gelation temperature and storage moduli (values at T_GEL_) of copolymer blends in water and in 0.9% NaCl and also with the presence of IgG (polymer concentration 25 wt%; IgG concentration 16.8 mg/mL).

Copolymer Mixture	T_GEL_ in H_2_O [°C]	T_GEL_ in NaCl [°C]	T_GEL_ in NaCl with IgG [°C]	Storage Modulus (E′) [kPa]	
				25%_NaCl_	25%_NaCl+IgG_
**P1/P3**	33.5	30.8	n.d.	1045.0	n.d.
**P2/P3**	32.9	30.5	19.5	968.0	1315.4
**P1/P4**	35.0	33.2	n.d.	2294.0	n.d.
**P2/P4**	33.4	30.5	29.4	550.0	700.0
**P2/P4/P6**	n.d.	32.4	28.5	1200.0	1345.0

n.d.—not determined.

**Table 4:** Gelation temperatures of modified copolymers and their blends and storage moduli (values at T**_GE_**_L_) of obtained gels (polymer concentration 25 wt%, IgG concentration 16.8 mg/mL).

Copolymer/Copolymer Mixture	T_GEL_ in NaCl [°C]	T_GEL_ in NaCl with IgG [°C]	Storage Modulus (E′) [kPa]	
			25%_NaCl_	25%_NaCl+IgG_
**P2M**	19.0	n.d.	1050.0	n.d.
**P4M**	28.0	n.d.	940.0	n.d.
**P2M/P4M**	26.3	24.4	842.0	950
**P2M/P4M/P6**	28.2/33.4 *	23.2 **	1750.0	n.d.

* T_GEL_ values from the two maxima obtained for the individual components of the blend; ** visual determination.

## Data Availability

The data presented in this study are available on request from the corresponding author.

## Supplementary Materials

The following supporting information can be downloaded at: https://www.mdpi.com/article/10.3390/ma17184472/s1, Figure S1. Functionalization of PLGA–PEG–PLGA to acryl–PLGA–PEG–PLGA–acryl copolymers. Figure S2. ^1^H NMR spectra made in CDCl_3_ of representative copolymers (a) **P2**, (b) modified **P2M**. Signals marked with * correspond to CDCl_3_, ** corresponds to residual DCM. Figure S3. UV-Vis spectra of copolymer **P2** before and after modification. Figure S4. Percentage weight loss during degradation of (a) **P1**/**P3**, (b) **P1**/**P4**, (c) **P2**/**P3**, (d) **P2**/**P4**, (e) **P2M**/**P4M**, and (f) **P2**/**P4**/**P6** copolymer blends in 0.9% NaCl.
