# Supplementary material for: Diverse Strategies to Develop Poly(ethylene glycol)–Polyester Thermogels for Modulating the Release of Antibodies
Source: Materials (Basel). 2024 Sep 12;17(18):4472. doi: 10.3390/ma17184472 (PMC11433636; doi:10.3390/ma17184472)
Supplement: Supplementary file 1 [file materials-17-04472-s001.zip › materials-3172409-supplementary.pdf]

Diverse strategies to develop PEG-polyester thermogels for modulating the release of antibodies

Daria Lipowska-Kur*, Łukasz Otulakowski, Urszula Szeluga, Katarzyna Jelonek, Alicja Utrata-Wesołek*

Centre of Polymer and Carbon Materials, Polish Academy of Sciences, M. Curie-Skłodowskiej 34, 41-819 Zabrze, Poland
* Correspondence: dlipowska@cmpw-pan.pl; autrata@cmpw-pan.pl; tel.: +48-32-271-60-77

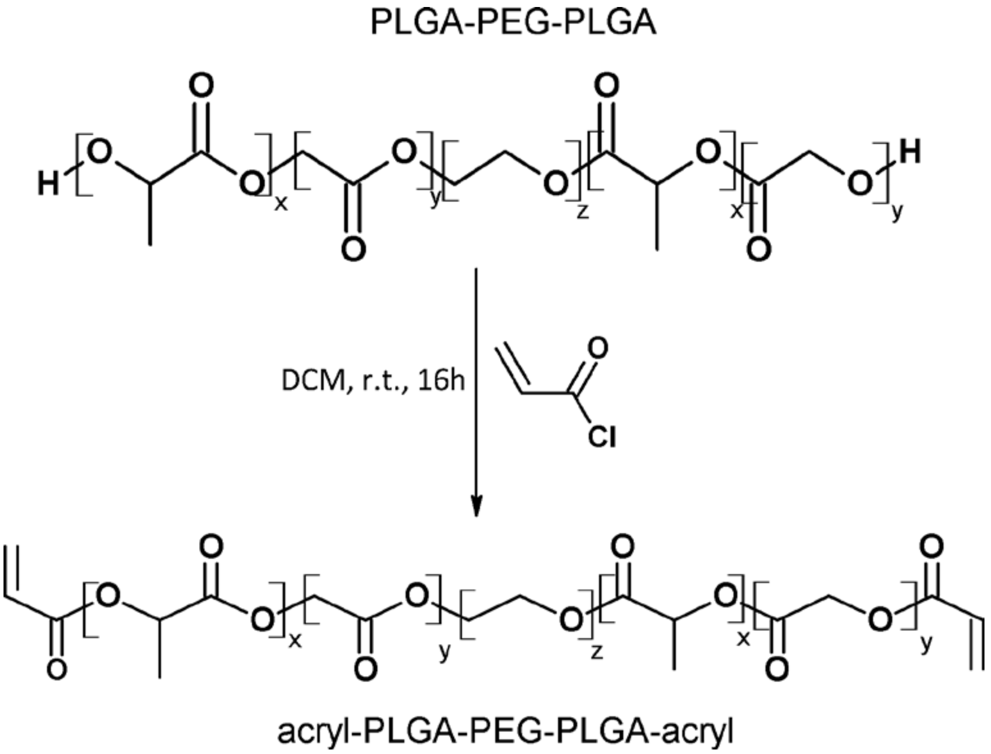

Figure S1. Functionalization of PLGA-PEG-PLGA to acryl-PLGA-PEG-PLGA-acryl copolymers.

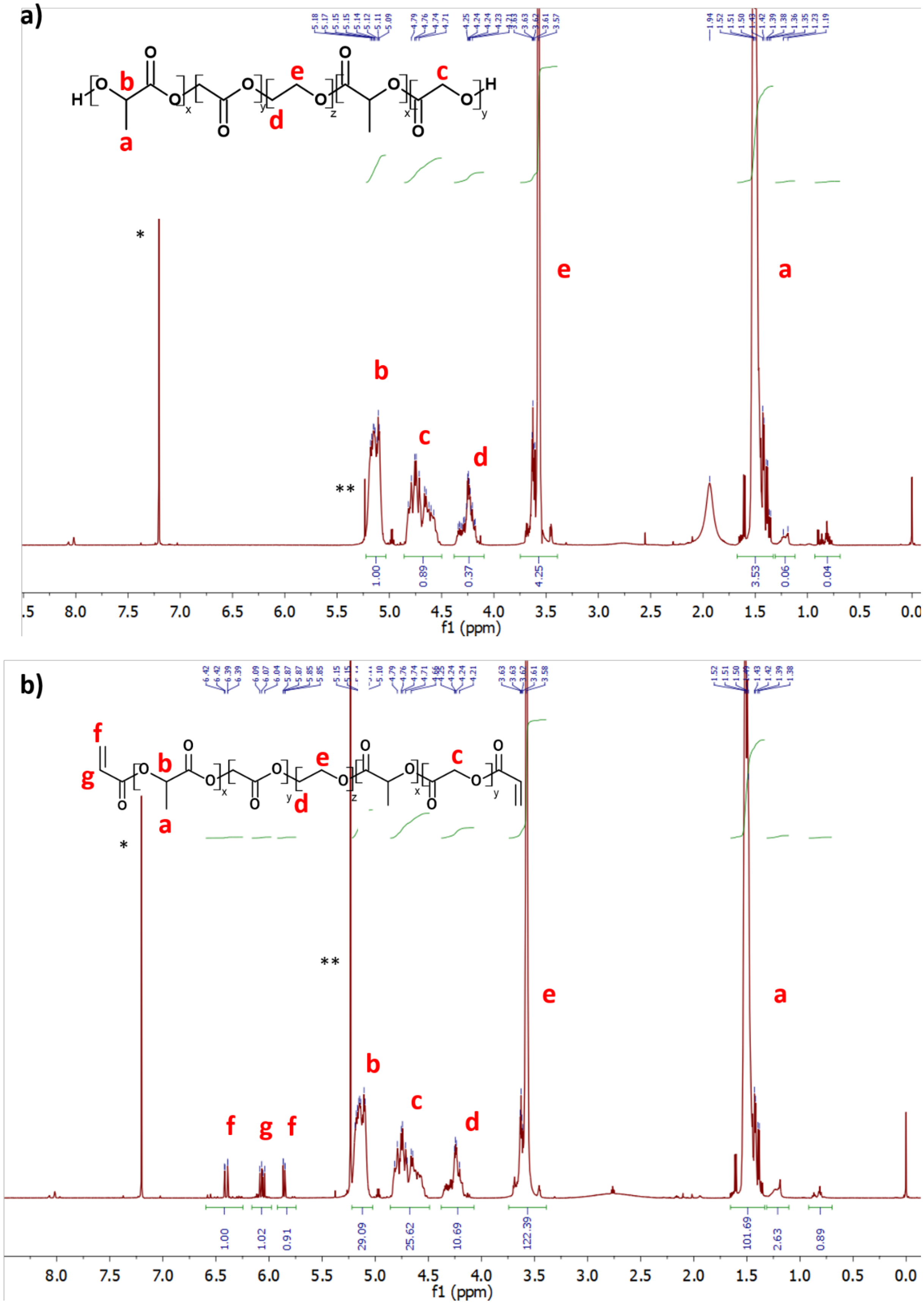

Figure S2. ^{1}H NMR spectra made in $CDCl_3$ of representative copolymers a) **P2**, b) modified **P2M**. Signals marked with * correspond to $CDCl_3$, ** correspond to residual DCM.

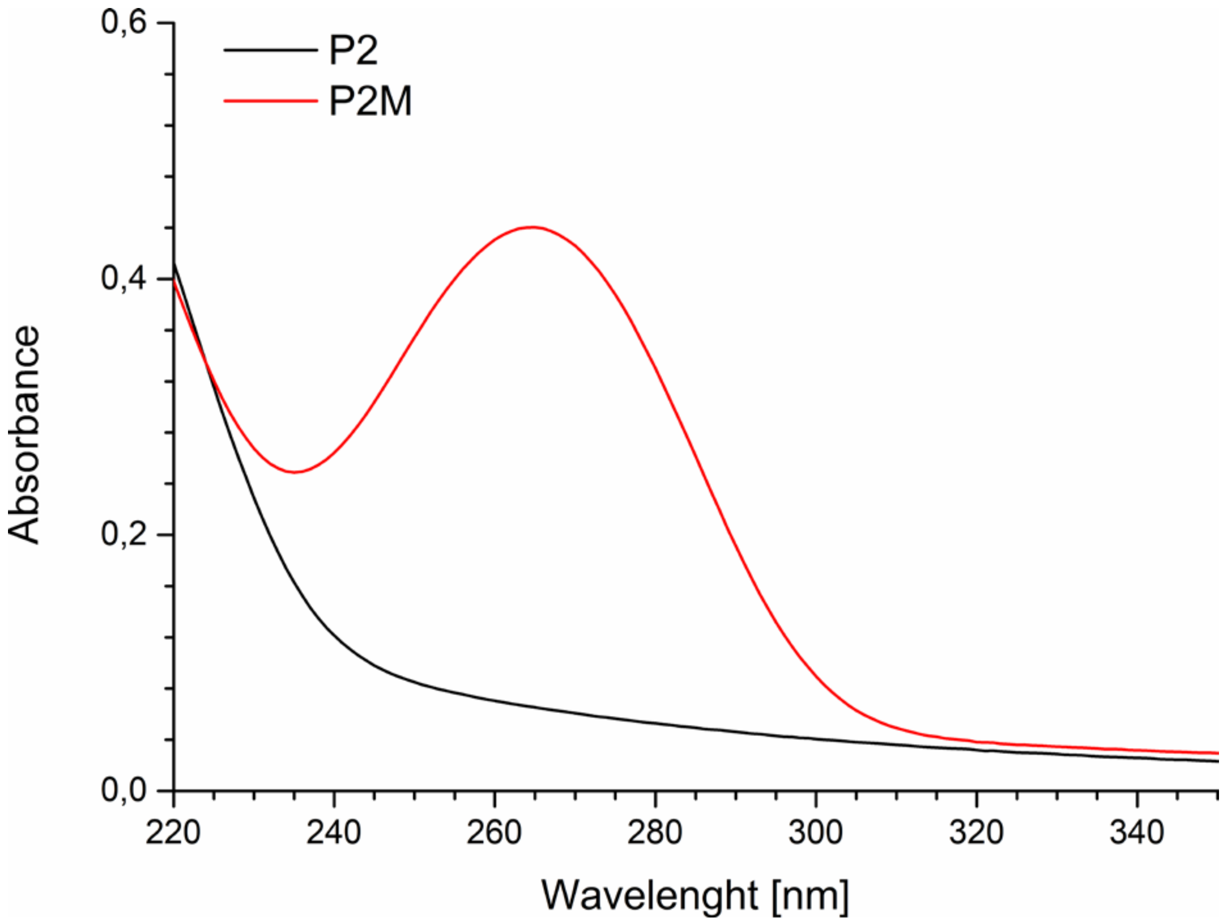

Figure S3. UV-Vis spectra of copolymer **P2** before and after modification.

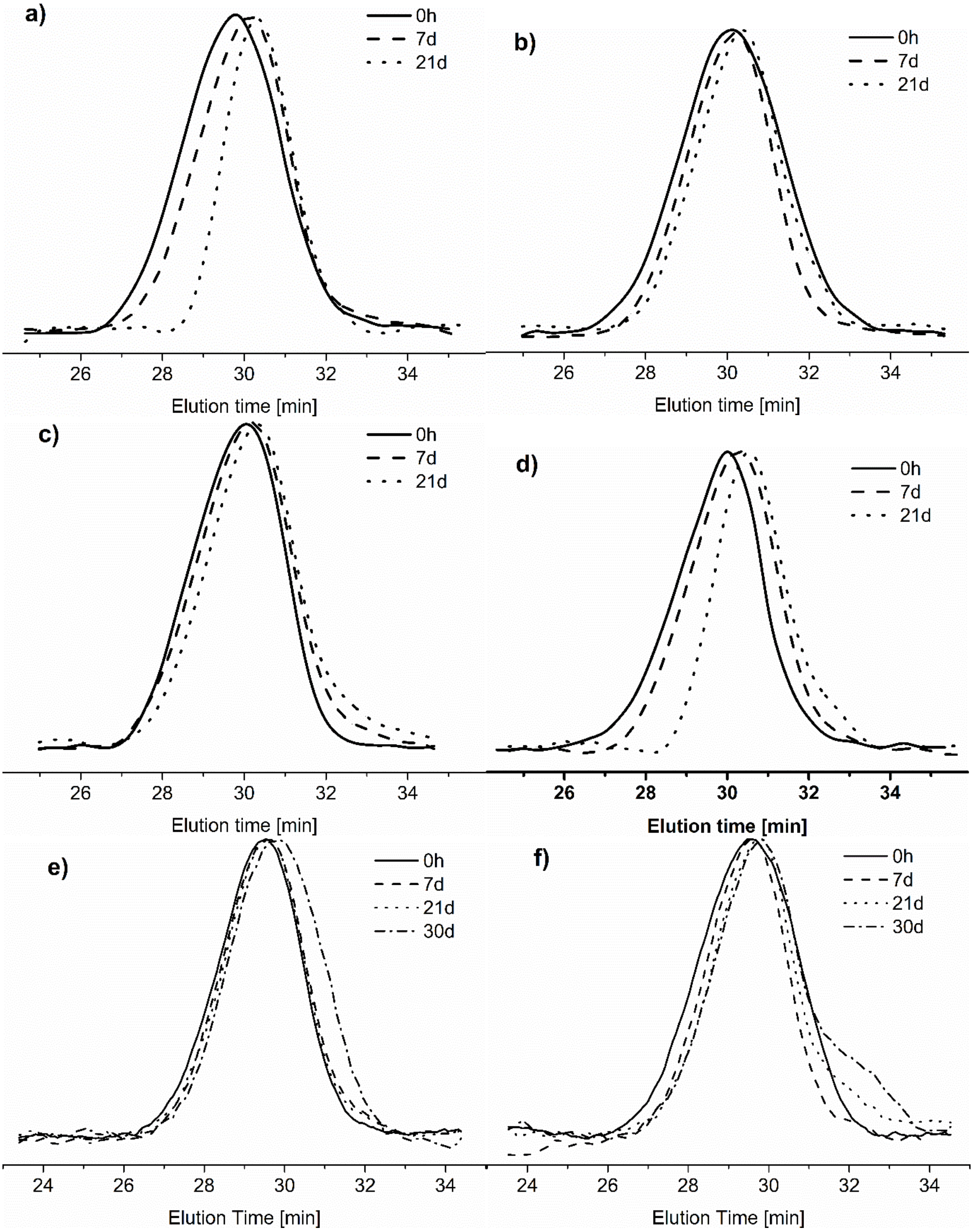

Figure S4. Percentage weight loss during degradation of (a) P1/P3, (b) P1/P4, (c) P2/P3, (d) P2/P4, (e) P2M/P4M and (f) P2/P4/P6 copolymer blends in 0.9% NaCl.
